# Resveratrol Propionate Ester Supplement Exerts Antihypertensive Effect in Juvenile Rats Exposed to an Adenine Diet via Gut Microbiota Modulation

**DOI:** 10.3390/nu16132131

**Published:** 2024-07-04

**Authors:** You-Lin Tain, Chi-I Chang, Chih-Yao Hou, Guo-Ping Chang-Chien, Shu-Fen Lin, Chien-Ning Hsu

**Affiliations:** 1 Division of Pediatric Nephrology, Kaohsiung Chang Gung Memorial Hospital, Kaohsiung 833, Taiwan; tainyl@cgmh.org.tw; 2 College of Medicine, Chang Gung University, Taoyuan 330, Taiwan; 3 Department of Biological Science and Technology, National Pingtung University of Science and Technology, Pingtung 912, Taiwan; changchii@mail.npust.edu.tw; 4 Traditional Herbal Medicine Research Center, Taipei Medical University Hospital, Taipei 110, Taiwan; 5 Department of Seafood Science, National Kaohsiung University of Science and Technology, Kaohsiung 811, Taiwan; chihyaohou@webmail.nkmu.edu.tw; 6 Institute of Environmental Toxin and Emerging-Contaminant, Cheng Shiu University, Kaohsiung 833, Taiwan; guoping@csu.edu.tw (G.-P.C.-C.); linsufan2003@gmail.com (S.-F.L.); 7 Super Micro Mass Research and Technology Center, Cheng Shiu University, Kaohsiung 833, Taiwan; 8 Center for Environmental Toxin and Emerging-Contaminant Research, Cheng Shiu University, Kaohsiung 833, Taiwan; 9 Department of Pharmacy, Kaohsiung Chang Gung Memorial Hospital, Kaohsiung 833, Taiwan; 10 School of Pharmacy, Kaohsiung Medical University, Kaohsiung 807, Taiwan

**Keywords:** short chain fatty acid, gut microbiota, resveratrol, chronic kidney disease, nitric oxide, propionate, hypertension

## Abstract

Resveratrol, acting as a prebiotic, and propionate, functioning as a postbiotic, hold promise for preventing hypertension in chronic kidney disease (CKD). Previously, we employed propionate to enhance the bioavailability of resveratrol through esterification, resulting in the production of a resveratrol propionate ester (RPE) mixture. In this study, we purified 3-O-propanoylresveratrol (RPE2) and 3,4′-di-O-propanoylresveratrol (RPE4) and investigated their protective effects in a juvenile rat adenine-induced CKD model. To this end, male Sprague Dawley rats aged three weeks (*n* = 40) were divided into five groups: control; CKD (rats fed adenine); CKRSV (CKD rats treated with 50 mg/L resveratrol); CDRPE2 (CKD rats treated with 25 mg/L RPE2); and CKRPE4 (CKD rats treated with 25 mg/L RPE 4). RPE2 and PRE4 similarly exhibited blood pressure-lowering effects comparable to those of resveratrol, along with increased nitric oxide (NO) availability. Furthermore, RPE2 and RPE4 positively influenced plasma short-chain fatty acid (SCFA) levels and induced distinct alterations in the gut microbial composition of adenine-fed juvenile rats. The supplementation of RPE2 and RPE4, by restoring NO, elevating SCFAs, and modulating the gut microbiota, holds potential for ameliorating CKD-induced hypertension.

## 1. Introduction

Hypertension is a significant complication and risk factor for the progression of chronic kidney disease (CKD) in children [1]. Early identification and treatment of hypertension can reverse early cardiovascular disease, thereby delaying CKD progression and improving both cardiovascular morbidity and mortality. The interplay between the kidneys and gut, known as the bidirectional kidney–gut axis, plays a pivotal role in CKD pathogenesis. The uremic environment contributes to gut microbiota dysbiosis, with gut microbial metabolites implicated in CKD progression and associated comorbidities, including hypertension [2].

CKD severity can be manipulated by altering adenine concentrations in the diet [3]. Our prior research revealed that juvenile rats treated with 0.5% adenine developed CKD, mirroring pediatric CKD in humans [4]. In this model, CKD-induced hypertension correlated with changes in gut microbiota composition, their metabolites, and decreased nitric oxide (NO) availability.

Prebiotics and postbiotics are widely employed therapies targeting gut microbiota, offering numerous health benefits [5,6]. Resveratrol is a naturally occurring polyphenol found in various plants and fruits, including grapes, berries, and peanuts [7,8]. It possesses a range of biological functions, such as antioxidant properties, anti-inflammatory effects, anti-obesogenic activity, and anti-atherosclerotic properties [7,8]. Similar to other polyphenols, resveratrol has been shown to enhance gut microbial composition in both human and animal studies [9,10], meeting the definition of prebiotics [11]. Despite its potential in addressing CKD and hypertension [7,8,12], limited availability hampers its clinical utility [13].

Postbiotic components include short-chain fatty acids (SCFAs) [14,15], which are major microbial metabolites [2]. SCFAs play crucial roles in the development of CKD and hypertension [2,14,15]. Propionate, a key SCFA, provides significant health benefits [16]. Previous studies have demonstrated the efficacy of postbiotic propionate in lowering blood pressure (BP) in animal models [17,18].

Our research, alongside others, has shown that esterification of resveratrol with SCFAs using Steglich reactions enhances its bioactivity and antioxidant capacity compared to resveratrol alone [19,20]. We introduced novel resveratrol propionate esters (RPEs), comprising resveratrol (~19.9%), RPE monoester (~45.8%), and RPE diester (~32.8%) [19]. Notably, RPE exhibits superior inhibition of oxidized low-density lipoprotein, a marker of oxidative stress from lipid oxidation, compared to resveratrol and other resveratrol-SCFA esters [19].

While some dietary supplements have demonstrated benefits in ameliorating hypertension [21,22,23], the potential BP-lowering effect of resveratrol-SCFA esters has only recently garnered attention and remains incompletely understood [24]. Our research aims to refine RPEs and assess their protective mechanisms compared to resveratrol for treating CKD-induced hypertension, utilizing a juvenile rat model with adenine-induced CKD.

## 2. Materials and Methods

### 2.1. Synthesis of Resveratrol Propionate Esters

A quantity of 60 g of trans-resveratrol (TCI Development Co., Ltd., Shanghai, China) was stirred with 80 mL of propanoic anhydride (ACROS, Morris Plains, NJ, USA) in 200 mL of pyridine for 8 h at room temperature, following the procedure outlined in a previous study [19]. The resulting reaction mixture was slowly added to ice-cooled water with continuous stirring and subsequently extracted three times with 500 mL of ethyl acetate. The combined ethyl acetate solution was subjected to sequential washing with 3 N HCl, saturated aqueous NaHCO_3_, and brine, followed by drying with Na_2_SO_4_. Upon evaporation of the solvent under reduced pressure, a mixture of RPE compounds weighing 52 g was obtained.

### 2.2. Purification of RPE Esters

**Figure 1:**
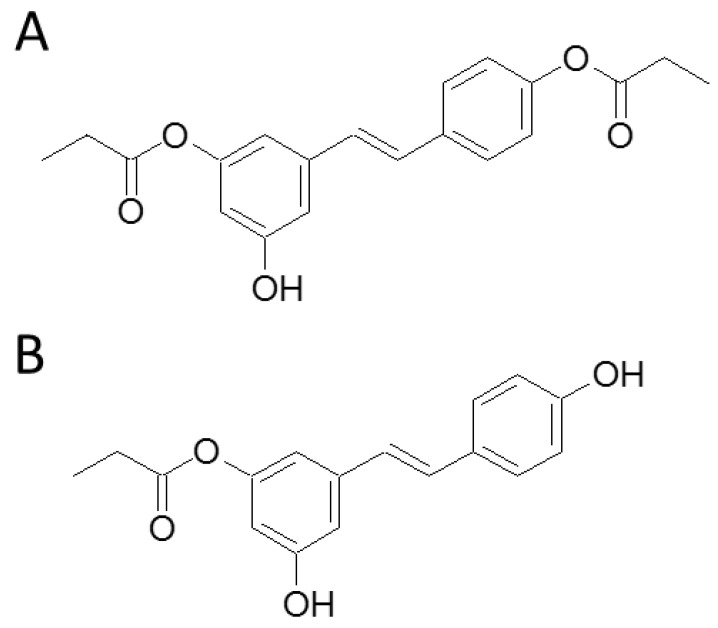
The chemical structures of (**A**) RPE2 (3-O-propanoylresveratrol) and (**B**) RPE4 (3,4′-di-O-propanoylresveratrol).

We proceeded with the purification and preparation of RPE esters [24]. In summary, the reaction mixture underwent chromatography on a silica gel column (7 × 120 cm), with elution using a CH_2_Cl_2_–acetone gradient of increasing polarity, followed by MeOH, resulting in 92 fractions. Based on thin-layer chromatography findings, these fractions were consolidated into 16 major fractions (1–16). Fraction 4 (7.6 g) from CH_2_Cl_2_–acetone (97:3) elution underwent further purification through a silica gel column (5 × 50 cm) using CH_2_Cl_2_–EtOAc (100:0–90:1) as the elution solvent, yielding 3,4′-di-O-propanoylresveratrol (3.2 g). Fraction 10 (8.6 g) from CH_2_Cl_2_–acetone (9:1) elution underwent additional purification through a silica gel column (5 × 50 cm) using CH_2_Cl_2_–EtOAc (85:1–5:1) to yield 14 fractions (10A–10N). Fraction 10E, after purification by crystallization from acetone, produced 3-O-propanoylresveratrol (3.6 g). Subsequent to the purification of the mixture of RPEs, we identified the structural types of 3-O-propanoylresveratrol (RPE2) and 3,4′-di-O-propanoylresveratrol (RPE4) and employed them in the subsequent experiments. Their chemical structures are presented in Figure 1.

### 2.3. Animal Model

In compliance with our Institutional Animal Care and Use Committee (permit # 2022081803), all animal experimentation was carried out with prior approval. Sprague Dawley (SD) rats were procured from BioLASCO Taiwan Co., Ltd. (New Taipei City, Taiwan). The research was conducted in an AAALAC-accredited animal facility, with an exclusive focus on male rats owing to their heightened vulnerability to hypertension at a younger age [25].

**Figure 2:**
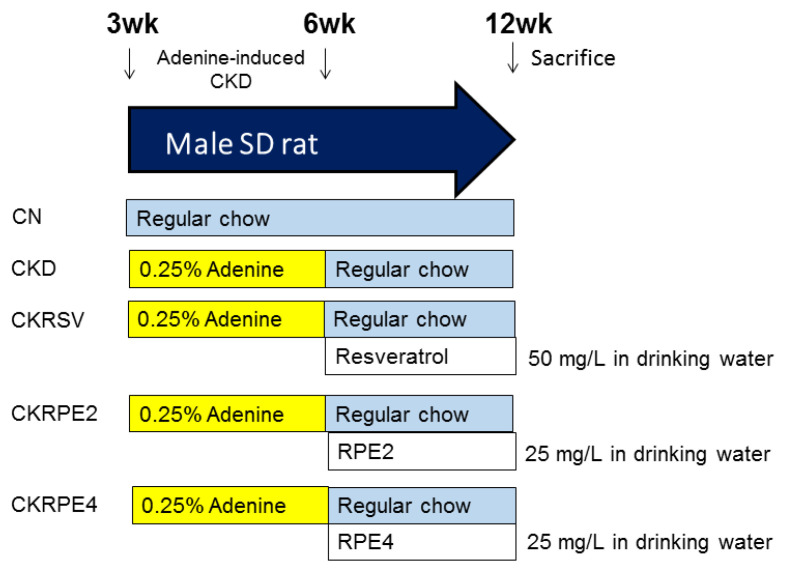
Experimental protocol used in the current study. CN, rats fed with regular chow; CKD, rats fed with 0.25% adenine; CKRSV, adenine-fed rats treated with resveratrol; CKRPE2, adenine-fed rats treated with RPE2; CKRPE4, adenine-fed rats treated with RPE4.

Figure 2 depicts the design of the animal study. At the age of 3 weeks, rats were divided into two groups: one group received regular feed (Fwusow Taiwan Co., Ltd., Taichung, Taiwan; 52% carbohydrates, 23.5% protein, 4.5% fat, 10% ash, and 8% fiber) (referred to as the CN group; *n* = 8), while the other was fed with 0.25% adenine chow (CKD group; *n* = 32) for 3 weeks. Provided with resveratrol at a concentration of 50 mg/L in their drinking water for 6 weeks (weeks 6–12) were a subset of adenine-treated CKD rats (CKRSV group). Another set of CKD rats received treatment with RPE2 (25 mg/L in drinking water, CKRPE2 group) or RPE4 (25 mg/L in drinking water, CKRPE4 group) for the same duration (weeks 6–12).

Using an indirect tail-cuff method (CODA, Kent Scientific Corp., Torrington, CT, USA) [24], BPs were determined. Prior to measurements, rats underwent a one-week acclimation period to a restraint box and tail-cuff inflation. Upon reaching 12 weeks of age, the rats were euthanized. The rats were anesthetized via intraperitoneal injection of ketamine (50 mg/kg body weight) and xylazine (10 mg/kg body weight), and euthanized by administering an overdose of pentobarbital via intraperitoneal injection. Fecal samples were collected in the morning prior to sacrifice by lifting the tail and twisting it towards the back to induce defecation. Later, collected fecal samples were frozen and placed into a −80 °C freezer. Heparinized blood samples were obtained and stored at −80 °C. Kidneys were perfused with cold phosphate-buffered saline, decapsulated, removed and weighed. Kidney cortex and medulla were separated, flash-frozen in liquid nitrogen and stored at −80 °C for later analysis.

### 2.4. Measurement of SCFAs by GC-MS

Utilizing gas chromatography–mass spectrometry (GC-MS) equipped with an automated sampler (7890B, Agilent Technologies, Santa Clara, CA, USA), the plasma concentrations of SCFAs, encompassing acetic acid (C2), propionic acid (C3), butyric acid (C4), valeric acid (C5), and hexanoic acid (C6) were subjected to analysis [24]. Utilizing a DB-FFAP column (30 cm × 0.25 mm, 0.25 µm; Agilent Technologies), we accomplished chromatographic separation. The injection volume was fixed at 1 µL with a split ratio of 5:1, and the injection temperature was held at 240 °C. To ensure precise quantification, 2-ethylbutyric acid was employed as the internal standard throughout the analytical procedure.

### 2.5. Measurement of NO Elements by HPLC

Arginine serves as the substrate for nitric oxide synthase (NOS), while both symmetric and asymmetric dimethylarginine (SDMA and ADMA) function as inherent inhibitors of NOS. The quantification of these NO-related elements in plasma was conducted through high-performance liquid chromatography (HPLC) using a system supplied by Agilent Technologies Inc. (HP series 1100, Santa Clara, CA, USA). In this method, fluorescence detection was employed for o-phthalic aldehyde derivatization, with 3-mercaptopropionic acid facilitating the process [24]. The calculation of the arginine-to-ADMA ratio was performed to signify the availability of NO [26].

### 2.6. Quantitative Real-Time Polymerase Chain Reaction

RNA extraction was carried out from the kidney cortex of each rat, followed by renal gene expression analyses of SCFA receptors using qPCR and the SYBR Green PCR Reagents kit (Qiagen, Valencia, CA, USA). The resulting data were subsequently normalized to the 18S rRNA (R18S) reference gene. Four SCFA receptors, specifically G protein-coupled receptor 41 (GPR41), GPR43, GPR109A, and olfactory receptor 78 (Oflr78), were subjected to scrutiny using primer established earlier [24]. Gene expression was relatively quantified using the comparative threshold cycle method.

### 2.7. Microbiome Analysis

Extracted from stool samples, DNA from the microbial community was processed for subsequent 16S rRNA sequencing, following established protocols [18]. For subsequent sequencing, a multiplexed SMRTbell library (PacBio, Menlo Park, CA, USA) was prepared by amplifying the full-length bacterial 16S rRNA gene using barcoded primers. The sequencing process followed this preparation. In the construction of a phylogenetic tree, the QIIME2 phylogeny fast tree utilized a set of sequences representing amplicon sequence variants (ASVs) [27,28]. The QIIME2 algorithm employed the Greengenes reference database for taxonomic assignments of microbial sequences. The sequence reads obtained underwent filtration to exclude low-quality reads. High-quality reads were screened for chimeras, which were subsequently removed. The number of non-chimeric reads per sample ranged from 12,167 to 76,201.

Utilizing Faith’s phylogenetic diversity (PD) index and the Shannon index, alpha-diversity analysis, which assesses microbiota richness and evenness within a single sample, was conducted. Beta-diversity analysis involved the application of principal coordinate analysis (PCoA) based on the unweighted UniFrac distance of the amplicon sequence variants (ASVs), along with analysis of similarities (ANOSIM). Using linear discriminant analysis effect size (LEfSe) analysis, we further identified meaningfully differential taxa, with emphasis on a linear discriminant analysis (LDA) score surpassing 4.

### 2.8. Statistics

The data are presented as means ± the standard error of the mean (SEM), and statistical significance was set at *p* < 0.05. One-way analysis of variance (ANOVA) was employed for statistical analyses. Post hoc multiple comparison tests were conducted using Tukey’s post hoc test, with significance determined at a threshold of *p* < 0.05. We used the Statistical Package of the Social Sciences software 22.0 (SPSS Inc., Chicago, IL, USA) to analyze all data.

## 3. Results

### 3.1. Body Weight, BP, and Renal Function

In the CKRSV group, one rat died right after receiving resveratrol treatment (Table 1). A comparative analysis revealed that the body weight (BW) in the CKD, CKRSV, and CKRPE4 groups was lower than that in the CN group. Remarkably, RPE2 treatment mitigated the BW loss induced by CKD. Notably, both kidney weight (KW) and the KW-to-BW ratio exhibited lower values in the control group compared to the other four groups. The increased KW-to-BW ratio was alleviated by resveratrol, RPE2, and RPE4 (Table 1).

**Figure 3:**
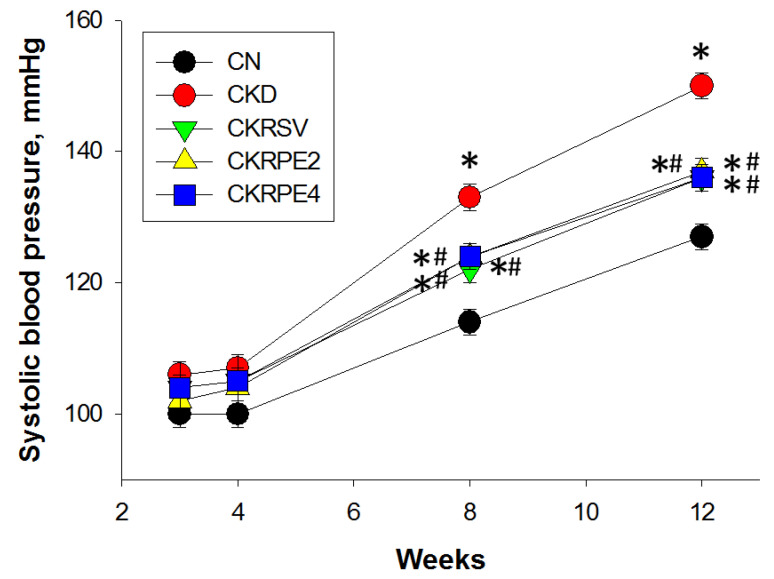
Effects of resveratrol and resveratrol propionate monoesters on systolic blood pressures in juvenile CKD rats from 3 to 12 weeks of age. *n* = 8/group; * *p* < 0.05 vs. CN; # *p* < 0.05 vs. CKD. CN, rats fed with regular chow; CKD, rats fed with 0.25% adenine; CKRSV, adenine-fed rats treated with resveratrol; CKRPE2, adenine-fed rats treated with RPE2; CKRPE4, adenine-fed rats treated with RPE4.

Figure 3 depicts a noteworthy increase in systolic BP in CKD rats by the age of 12 weeks, commencing at eight weeks of age. However, this elevation in systolic BP was significantly mitigated in the CKRSV, CKRPE2, and CKRPE4 groups. Concurrently, an elevation in plasma creatinine (Cr) levels was noted in CKD rats (Table 1). Intriguingly, treatment with RPE2 alone partially ameliorated the elevated Cr levels at 12 weeks of age (Table 1).

### 3.2. NO Pathway

Following the observed benefits of resveratrol and RPE2, and RPE4 in combating CKD-induced hypertension, our subsequent focus was on assessing the protective mechanisms across the four CKD groups. The beneficial effects of resveratrol on developmental programming of hypertension have been associated with an enhancement of NO availability [12]. We, hence, investigated whether resveratrol, RPE2, or RPE4 has a protective role on NO pathway (Table 2). Plasma concentrations of arginine exhibited no significant differences among the four groups. Notably, resveratrol demonstrated a reduction in plasma ADMA concentrations in the CKRSV group compared to the CKD group. Similarly, treatment with RPE2 or RPE4 increased NO availability, indicated by a decrease in plasma ADMA and SDMA, along with an elevation in the arginine-to-ADMA ratio in the CKRPE2 and CKRPE4 groups.

### 3.3. SCFAs and Their Receptors

Subsequently, we assessed the concentrations of SCFAs in the plasma and examined their receptors in the kidneys. Table 3 indicates that the CKRP2 group exhibited elevated plasma concentrations of acetic acid, propionic acid, and hexanoic acid compared to the CKD group. Plasma concentrations of propionic acid, butyric acid, and hexanoic acid were higher in the CKRPE4 group than those in the CKD group.

**Figure 4:**
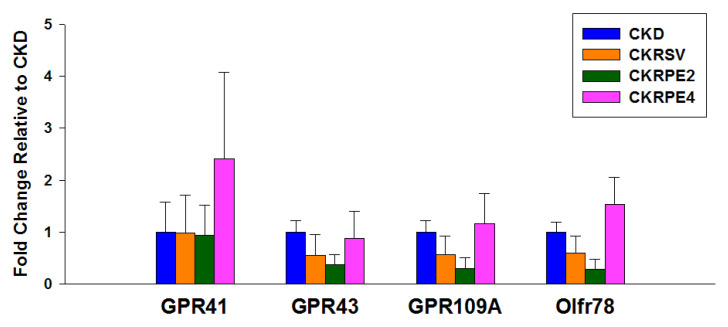
The mRNA expression of short chain fatty acid (SCFA) receptors in rat kidneys, including G protein-coupled receptor 41 (GPR41), GPR43, GPR109A, and olfactory receptor 78 (Oflr78). *n* = 7/group. CKD, rats fed with 0.25% adenine; CKRSV, adenine-fed rats treated with resveratrol; CKRPE2, adenine-fed rats treated with RPE2; CKRPE4, adenine-fed rats treated with RPE4.

Considering that SCFAs regulate BP through their receptors [10], we proceeded to conduct a more in-depth analysis of the mRNA expression of SCFA receptors in the kidneys. Figure 4 illustrates that renal expression of SCFA receptors did not differ across the four groups.

### 3.4. Gut Microbiota Compositions

**Figure 5:**
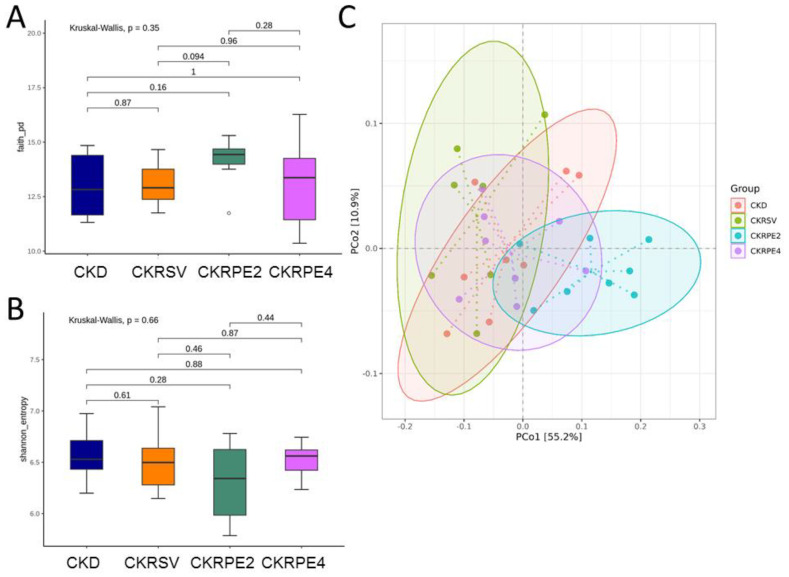
Evaluation of gut microbial community α-diversity among the four groups is illustrated in this figure, with (**A**) Faith’s phylogenetic diversity (PD) index and (**B**) the Shannon index. Furthermore, (**C**) bacterial β-diversity analysis was performed using principal coordinate analysis (PCoA) based on unweighted UniFrac distance. Each dot on the PCoA plot represents the microbiota of an individual sample, and the color of each dot corresponds to the metadata specific to that sample. *n* = 7–8/group. CKD, rats fed with 0.25% adenine; CKRSV, adenine-fed rats treated with resveratrol; CKRPE2, adenine-fed rats treated with RPE2; CKRPE4, adenine-fed rats treated with RPE4.

Illustrated in Figure 4 is the absence of differences in α-diversity among the four groups, as represented by the Faith’s PD index (Figure 5A) and the Shannon index (Figure 5B). Concerning β-diversity, the PCoA indicated a complete differentiation of microbial communities from the four groups into distinct clusters (Figure 5C). Additionally, the overall dissimilarity between the grouped communities was assessed by ANOSIM. The ANOSIM test revealed statistically significant differences among each group (*p* < 0.01), with the exception that the CKD group did not differ from the CKRSV group (*p* = 0.361).

**Figure 6:**
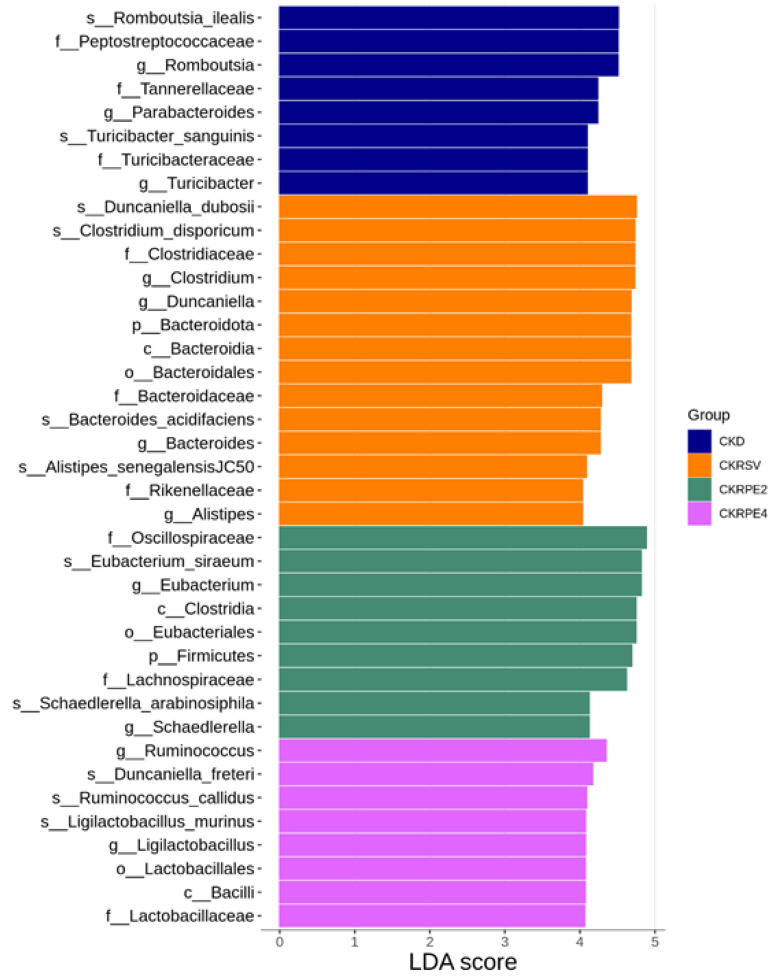
Set at a linear discriminant analysis (LDA) score greater than 4, the linear discriminant analysis effect size (LEfSe) was employed to compare differentially abundant taxa among the four groups. Different taxonomic levels of microbes are given reaching from phylum down to genuslevel. Each name is preceded by a letter giving the rank: p = phylum, c = class, o = order, f = family, g = genus, s = species. CKD, rats fed with 0.25% adenine; CKRSV, adenine-fed rats treated with resveratrol; CKRPE2, adenine-fed rats treated with RPE2; CKRPE4, adenine-fed rats treated with RPE4.

As shown in Figure 6, we utilized LEfSe analysis to identify taxonomic differences significantly abundant across the four groups. Notably, an upsurge in the abundance of *Clostridium* and *Bacteroides* genera was observed with resveratrol treatment. Treatment with RPE monoester 2 resulted in an increased proportion of *Eubacterium*. Furthermore, the CKRPE4 group exhibited heightened levels of *Ruminococcus* and *Ligilactobacillus* genera (Figure 6). Particularly noteworthy is the increased prevalence of *Ligilactobacillus murinus* species within the CKRPE4 group, accompanied by its corresponding genus, family, order, and class.

**Figure 7:**
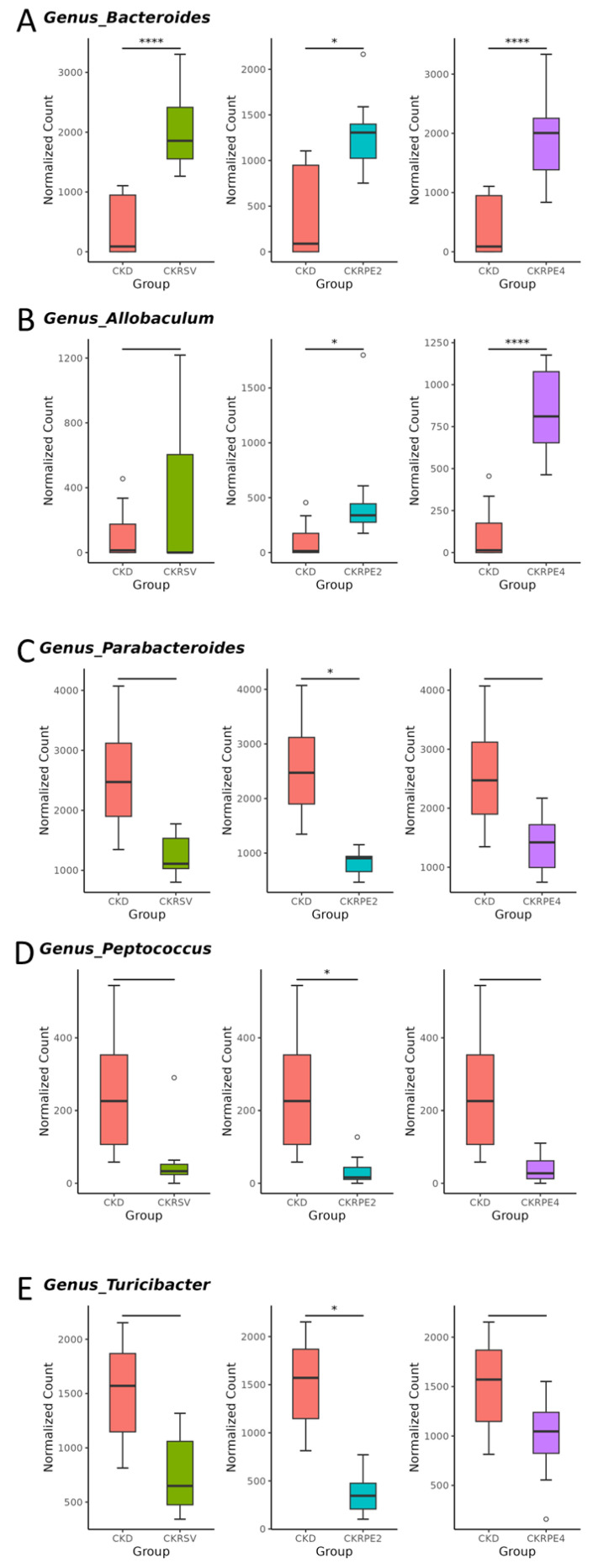
Genus-based comparison among the four groups, showcasing the relative abundance of (**A**) *Bacteroides*, (**B**) *Allobaculum*, (**C**) *Parabacteroides*, (**D**) *Peptococcus*, and (**E**) *Turicibacter*. Outliers are denoted by dots. * *p* < 0.05; **** *p* < 0.001. CKD, rats fed with 0.25% adenine; CKRSV, adenine-fed rats treated with resveratrol; CKRPE2, adenine-fed rats treated with RPE2; CKRPE4, adenine-fed rats treated with RPE4.

At the genus level, resveratrol, as well as RPE monomers 2 and 4, demonstrated an enhancement in the abundance of *Bacteroides* (Figure 7A). RPE monomers, specifically in the CKRPE2 and CKRPE4 groups, exhibited an augmentation in *Allobaculum* compared to the CKD group (Figure 7B). Moreover, a trend was observed where the CKD group tended to possess a higher abundance of *Parabacteroides*, *Peptococcus*, and *Turicibacter* genera compared to the other three groups (Figure 7C–E), although significance was only reached in the CKRPE2 group.

## 4. Discussion

For the first time, this study unveils the potential of RPEs in ameliorating CKD-induced hypertension within a juvenile rat model of CKD. Our pivotal findings are delineated as follows: (1) the manifestation of hypertension in adenine-treated juvenile CKD rats can be mitigated through dietary supplementation with resveratrol, RPE2 and RPE4; (2) the beneficial effects of RPE2 and RPE4 align with an augmentation in NO availability, evidenced by diminished levels of ADMA and SDMA, accompanied by an elevated arginine-to-ADMA ratio; (3) the favorable impacts of RPE2 and RPE4 correlate with heightened plasma SCFA levels; (4) RPE2 and RPE4 induce distinctive alterations in the gut microbial compositions of CKD rats; (5) the protective influence of RPE2 is connected to a greater genera proportion of *Eubacterium*, *Bacteroides*, and *Allobaculum*, coupled with a decrease in *Parabacteroides*, *Peptococcus*, and *Turicibacter*; and (6) RPE4 shields CKD rats from hypertension, concomitant with elevated quantities of the genera *Ruminococcus*, *Ligilactobacillus*, *Bacteroides*, and *Allobaculum*.

Resveratrol is widely believed to promote cardiovascular health; however, the results of meta-analyses have been inconclusive [29,30]. Given the limited bioavailability of resveratrol, the current study prepared resveratrol derivatives (RPEs) to enhance its efficacy. In line with previous research emphasizing the health-promoting benefits of resveratrol derivatives, acknowledged for their dual role as prebiotics and natural antioxidants [8,11,12], our findings contribute to this body of knowledge. This investigation specifically delves into the comparison of BP-lowering effects among resveratrol, RPE2, and RPE4 using a juvenile rat CKD model. Significantly, we present a novel observation that low-dose RPE2 and RPE4 (25 mg/L) confer protection against hypertension and the increased KW-to-BW ratio in juvenile CKD rats, paralleling the protective effects of resveratrol at a dose of 50 mg/L. Notably, it is essential to highlight that among the RPE esters, only RPE2 demonstrated attenuation of increased Cr levels and body weight loss. The beneficial effects attributed to RPE stem from its composition of pristine resveratrol and diverse ester derivatives [19]. Nevertheless, the precise ester derivatives responsible for these effects remain unclear. This study builds upon our earlier investigations by indicating that RPE2 and RPE4 both yield comparable BP-lowering effects to resveratrol, while RPE2 exhibits superior protection in decreasing Cr levels. While the total quantity of compounds administered in both RPE2 and RPE4 remains equal, there is variation in the amount of resveratrol present in each of the two esters. Whether this difference might have an impact on the obtained results requires further evaluation. Additional studies are required to assess the bioavailability of RPE2 and RPE4 to understand their pharmacokinetic profile and optimize their therapeutic efficacy.

Several mechanisms underlying hypertension, aligning with or correlating to the favorable effects of resveratrol derivative supplementation, were characterized. Initially, the advantageous outcomes of all three treatments are linked to an increase in NO availability. The observed reduction in ADMA levels, which contribute to NO deficiency and are implicated in CKD-associated hypertension [31], with all three resveratrol derivatives may be linked to their BP-lowering effects. Furthermore, RPE2 and RPE4 exhibit the capability to decrease SDMA levels and elevate the arginine-to-ADMA ratio, potentially further enhancing the NO pathway. Previous research suggests that resveratrol supplementation reduces BP primarily through the enhancement of NO production, which is mainly associated with the upregulation of endothelial nitric oxide synthase (eNOS) expression, increased eNOS phosphorylation, and the mitigation of eNOS uncoupling [32]. Our study further advances prior work by showing that RPE derivatives aid in improving BP by diminishing levels of NOS inhibitors, specifically ADMA and SDMA.

An additional safeguarding mechanism of RPE derivatives against CKD-induced hypertension may stem from an increase in SCFAs. Our findings regarding SCFAs corroborate prior reports demonstrating the BP-lowering actions of SCFA supplementation, including acetate, propionate, and butyrate [33]. Notably, RPE2 enhances acetic acid in addition to propionic acid, while RPE4 increases butyric acid. These results strongly suggest that the beneficial role of RPE derivatives is, at least partially, connected to the regulation of SCFAs.

The protective effects of RPE align with an evolving understanding of resveratrol acting as a prebiotic for the gut microbiota. Despite shaping a distinct microbiome composition from CKD, both RPE2 and RPE4 exhibit no difference in α-diversity between these groups, akin to resveratrol. Recently recognized probiotics such as *Bacteroides*, *Allobaculum*, *Clostridium*, and *Eubacterium* [34], as well as lactic acid bacteria such as *Ruminococcus* and *Ligilactobacillus* promoted by a plant-based high-fiber diet for human health [35], have garnered attention. *Ligilactobacillus*, in particular, has emerged as a notable probiotic [36]. Our data indicate that all three treatments enhance the abundance of *Bacteroides*. Resveratrol treatment results in an increase in the genus *Clostridium*. RPE2 elevates quantities of genera *Eubacterium* and *Allobaculum*, while RPE4 increases the abundance of genera *Allobaculum*, *Ruminococcus*, and *Ligilactobacillus*. This suggests that the beneficial effects of resveratrol and its RPE derivatives may be linked to an increase in these beneficial microbes.

In line with studies involving hypertensive individuals and animals [37,38,39], we note negative correlations between BP and the high abundance of the genera *Ruminococcus*, *Clostridium*, and *Bacteroides*. Conversely, positive associations are observed with increased abundance of *Parabacteroides*, *Peptococcus*, and *Turicibacter*. Additionally, we find that RPE 4 augments the species *Ligilactobacillus murinus*, along with its corresponding genus and class, aligning with its BP-lowering effect. This finding is consistent with prior research suggesting a correlation between low abundance of *Ligilactobacillus murinus* and hypertension [36]. Our results raise the possibility that the beneficial actions of RPE2 and RPE4 may be linked to their abilities to alter taxa related to hypertension.

While our study contributes valuable insights, it comes with certain limitations. Firstly, the tested doses of RPEs may not comprehensively elucidate their dose-dependent effects, hindering a satisfactory comparison of effectiveness with resveratrol. Future studies with varied doses are warranted to determine the ideal dose for clinical translation. Secondly, we confined RPEs to the CKD group based on prior indications that resveratrol derivatives pose no adverse effects on normal controls [19,40,41]. However, further investigation is imperative to assess the safety of RPEs for potential pharmaceutical use rather than mere dietary supplementation [42]. Thirdly, our analysis unveils associations between gut microbiota compositions, SCFAs, and the beneficial effects of RPEs against hypertension and kidney dysfunction in a juvenile CKD rat model. However, further exploration is required to establish the causal relationship, with additional studies needed to assess microbial function and identify specific bacterial strains targeting SCFAs to substantiate the causal role of the gut microbiota. Lastly, the sex-dependency or model-dependency of the BP-lowering effects of RPEs may require verification through additional studies with both sexes in a second CKD model to corroborate our conclusions.

## 5. Conclusions

In conclusion, we enhanced the bioactivity of resveratrol and incorporated the health benefits of propionate by preparing resveratrol propionate esters. Two specific derivatives, RPE2 and RPE4, were purified and demonstrated protective effects in a juvenile rat model of adenine-induced CKD. These beneficial effects were associated with increased NO availability, elevated SCFA levels, and alterations in the gut microbiota. The supplementation of RPE holds promise for optimizing hypertension induced by CKD and expanding the clinical utility of resveratrol-based natural products. This is attributed to its ability to improve the bioavailability of resveratrol.

## Figures and Tables

**Table 1:** Weights, BP, and plasma creatinine level.

Groups	CN	CKD	CKRSV	CKRPE2	CKRPE4
Mortality (%)	0	0	12.5	0	0
Body weight (BW) (g)	334 ± 7	243 ± 13 *	289 ± 5 *	307 ± 7 #	286 ± 6 *
Left kidney weight (KW) (g)	1.14 ± 0.04	2.12 ± 0.07 *	1.70 ± 0.06 #*	1.98 ± 0.1 *	1.67 ± 0.05 *#
KW-to-BW ratio (g/kg)	3.4 ± 0.1	8.9 ± 0.1 *	5.9 ± 0.1 *#	6.4 ± 0.1 *#	5.8 ± 0.1 *#
Systolic BP (mmHg)	127 ± 1	150 ± 2 *	136 ± 1 #	137 ± 1 #	136 ± 1 #
Diastolic BP (mmHg)	86 ± 2	99 ± 2 *	86 ± 3 #	91 ± 2 #	87 ± 2 #
Creatinine (μM)	16.4 ± 0.5	23.2 ± 1.2 *	21.5 ± 0.8 *	19.1 ± 1 *#	20.8 ± 0.8 *

*n* = 8/group; * *p* < 0.05 vs. CN; # *p* < 0.05 vs. CKD; BP = blood pressure. CN, rats fed with regular chow; CKD, rats fed with 0.25% adenine; CKRSV, adenine-fed rats treated with resveratrol; CKRPE2, adenine-fed rats treated with RPE2; CKRPE4, adenine-fed rats treated with RPE4.

**Table 2:** NO-related elements.

Groups	CKD	CKRSV	CKRPE2	CKRPE4
Arginine, μM	203.4 ± 15.1	202.6 ± 8.3	227.1 ± 9.4	233.2 ± 21.5
ADMA, μM	2.71 ± 0.08	2.42 ± 0.07 *	2.12 ± 0.1 *	2.3 ± 0.04 *
SDMA, μM	2.84 ± 0.1	2.58 ± 0.09	2.38 ± 0.08 *	2.47 ± 0.06 *
Ratio of arginine-to-ADMA	75.6 ± 5.3	82.1 ± 2	105.4 ± 8.5 *	100.8 ± 12 *

*n* = 7/group. * *p* < 0.05 vs. CKD. CKD, rats fed with 0.25% adenine; CKRSV, adenine-fed rats treated with resveratrol; CKRPE2, adenine-fed rats treated with RPE2; CKRPE4, adenine-fed rats treated with RPE4.

**Table 3:** Plasma concentrations of SCFAs.

Groups	CKD	CKRSV	CKRPE2	CKRPE4
Acetic acid, ng/mL	561.1 ± 31.3	643.5 ± 19.4	779.5 ± 41.6 *	584.2 ± 34.5
Propionic acid, ng/mL	4.92 ± 0.17	4.61 ± 0.14	7.16 ± 0.23 *	7.46 ± 0.52 *
Butyric acid, ng/mL	9.79 ± 1.21	8.14 ± 0.41	10.81 ± 0.92	18.04 ± 0.78 *
Valeric acid, ng/mL	1.93 ± 0.08	1.94 ± 0.03	2.11 ± 0.07	1.92 ± 0.04
Hexanoic acid, ng/mL	16.14 ± 0.73	17.34 ± 0.43	21.07 ± 0.87 *	19.67 ± 0.73 *

*n* = 7/group. * *p* < 0.05 vs. CKD. CKD, rats fed with 0.25% adenine; CKRSV, adenine-fed rats treated with resveratrol; CKRPE2, adenine-fed rats treated with RPE2; CKRPE4, adenine-fed rats treated with RPE4.

## Data Availability

Data are contained within the article.
